# Supplementary material for: Reduction of exposure to simulated respiratory aerosols using ventilation, physical distancing, and universal masking
Source: Indoor Air. 2022 Feb 22;32(2):e12987. doi: 10.1111/ina.12987 (PMC8988470; doi:10.1111/ina.12987)
Supplement: Supplementary file 1 — Supplementary Material [file INA-32-0-s001.docx]

Reduction of exposure to simulated respiratory aerosols using ventilation, physical distancing, and universal masking

Jayme P. Coyle, Raymond C. Derk, William G. Lindsley, Theresa Boots, Francoise M. Blachere, Jeffrey S. Reynolds, Walter G. McKinney, Erik W. Sinsel, Angela R. Lemons, Donald H. Beezhold, and John D. Noti

Health Effects Laboratory Division, National Institute for Occupational Safety and Health, Centers for Disease Control and Prevention, Morgantown, West Virginia, USA

Corresponding Author

Dr. William G. Lindsley

National Institute for Occupational Safety and Health (NIOSH)

1000 Frederick Lane, M/S 4020

Morgantown, WV 26508-5402

Email: [wlindsley@cdc.gov](mailto:wlindsley@cdc.gov)


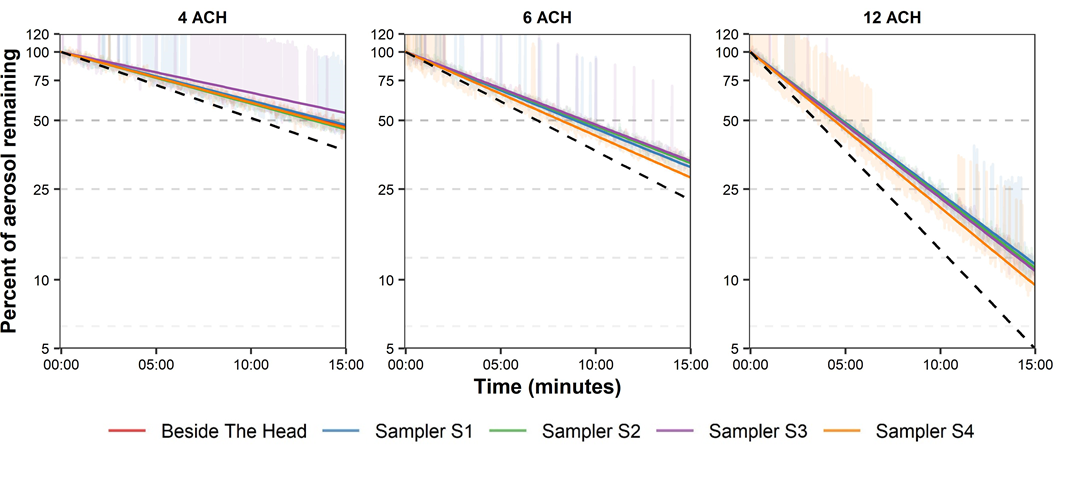


Supplemental Figure S1. Chamber Aerosol Decay Curves for each Grimm Optical Particle Counter. NaCl particles were produced with the TSI Particle Generator after particles from the smallest three bins were measured in real-time for 15 minutes at the examined ventilation rates. The exponential decay curves are overlain (solid lines) with theoretical decay rates (dotted lines) for a well-mixed room. ACH = Air changes per hour.


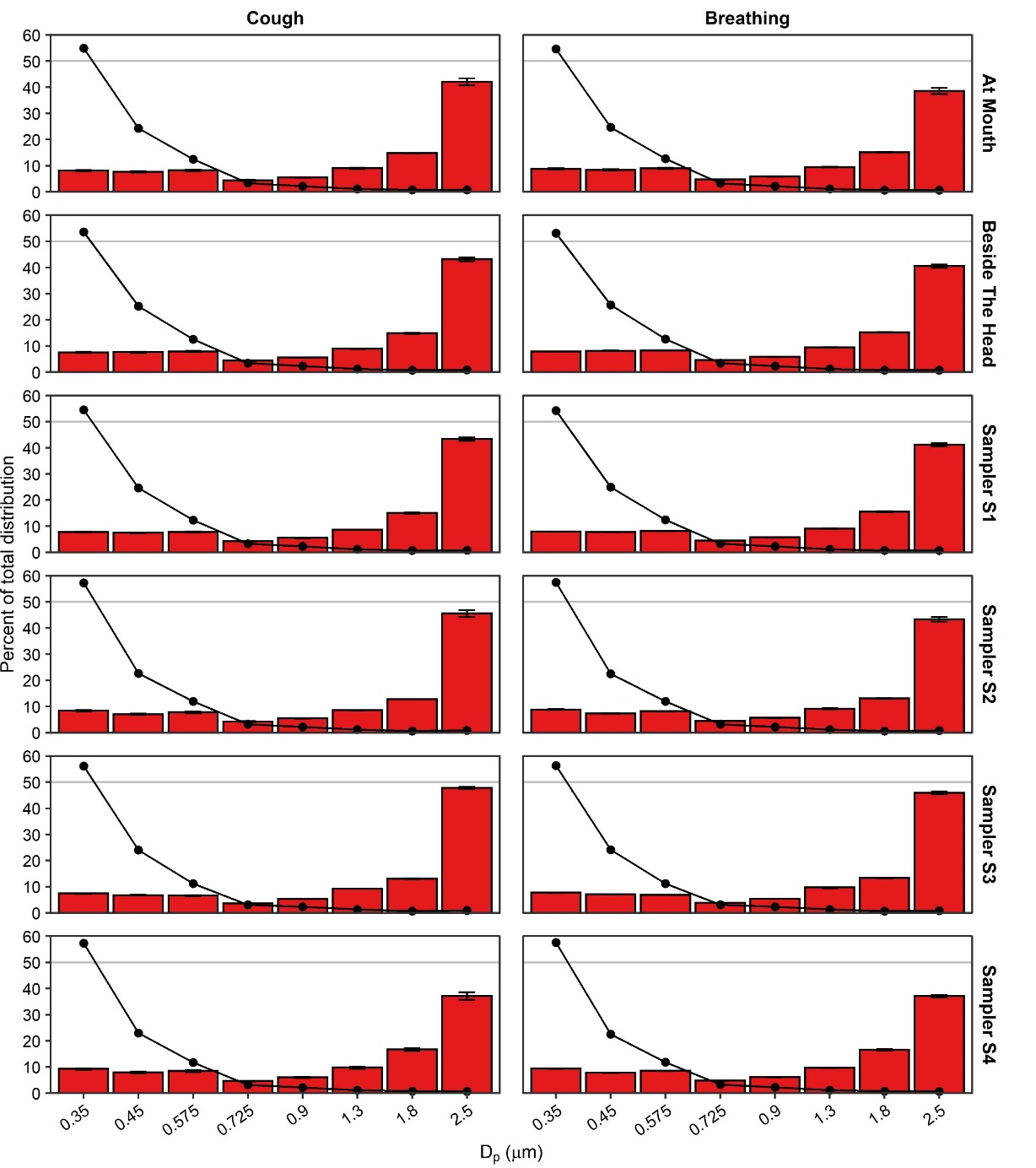


Supplemental Figure S2. OPC-stratified bin-specific particle distributions. Bin-specific particle distributions as determined by mass (bars) and by number of particles (line). The median particle diameter (Dp) indicates the bin. Results are the arithmetic mean ± standard deviation of three independent experiments. Error bars for the number of particles (line) too small to visualize. A horizontal line at 50% was overlain for reference.


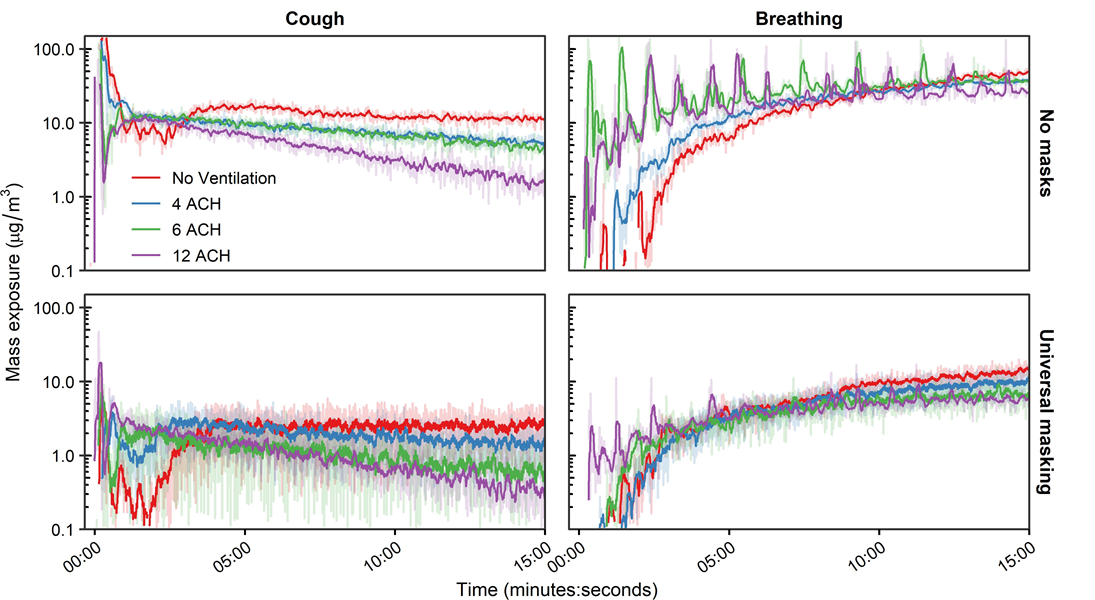
Supplemental Figure S3. Aerosol Mass Exposure of the Recipient. Mass exposure concentration time curves for a single cough and breathing, masking status, and ventilation for the 0.9 m physical distance. Results are the arithmetic mean of three independent experiments. ACH = Air changes per hour.


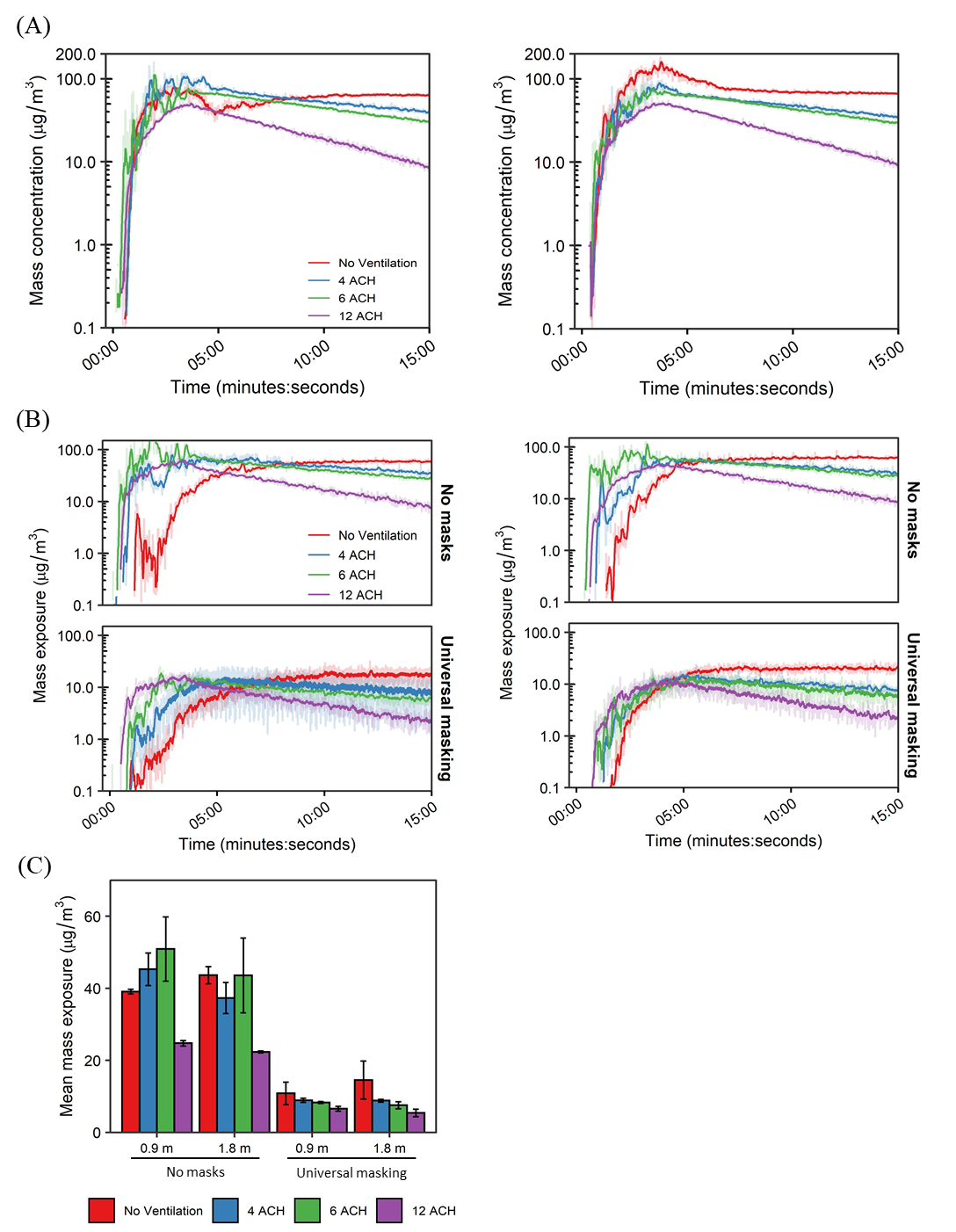


Supplemental Figure S4. Aerosol Mass Exposure of the Recipient in Short-term Aerosol Generation Tests. A) Area sampler time-concentration curves for the 0.9 m (Left) and 1.8 m (Right) physical distance. B) Time-concentration curves of aerosol exposure at the mouth of the recipient across the masking and ventilation. The curves for the 0.9 m (Left) and 1.8 m (Right) physical distance are presented for comparison. C) Mean mass exposure over the 15-minute simulation period derived from the time curves. Results are the arithmetic mean ± standard deviation of three independent experiments. ACH = Air changes per hour.


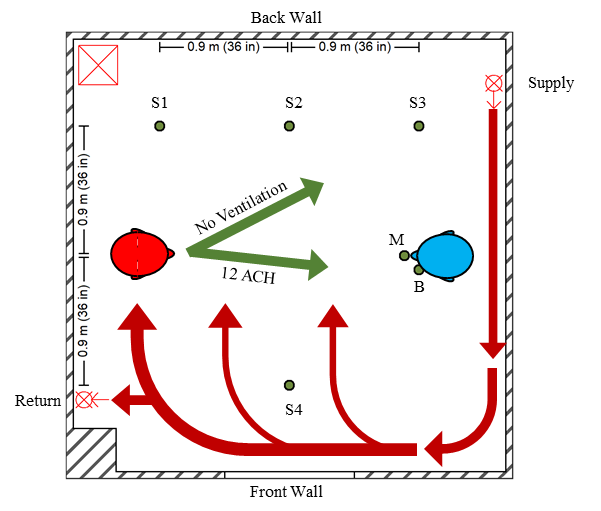


Supplemental Figure S5. Qualitative Air Flow Patterning within the Environmental Chamber.

Diagram of environmental chamber setup showing positions of the aerosol source simulator (red), recipient simulators (blue; position adjustable between 0.9 m and 1.8 m), and optical particle counters (green dots) for area measurements (S1–4) and personal breathing zone measurements at the mouth (M) and beside the head (B) of the recipient. The room return and supply for the HEPA system are each shown with a circle and “X”. The HEPA filter and blower unit are demarcated by the red square containing an “X”. Green arrows designate the general direction of air flow seen when placing the fog generator at the mouth of the source simulator when the simulator was not coughing or breathing. Red arrows designate the general air flow pattern from the ventilation system into the chamber at 12 ACH. The thin arrows denote the air flow pattern occurred primarily along the floor of the environmental chamber. ACH = Air changes per hour.


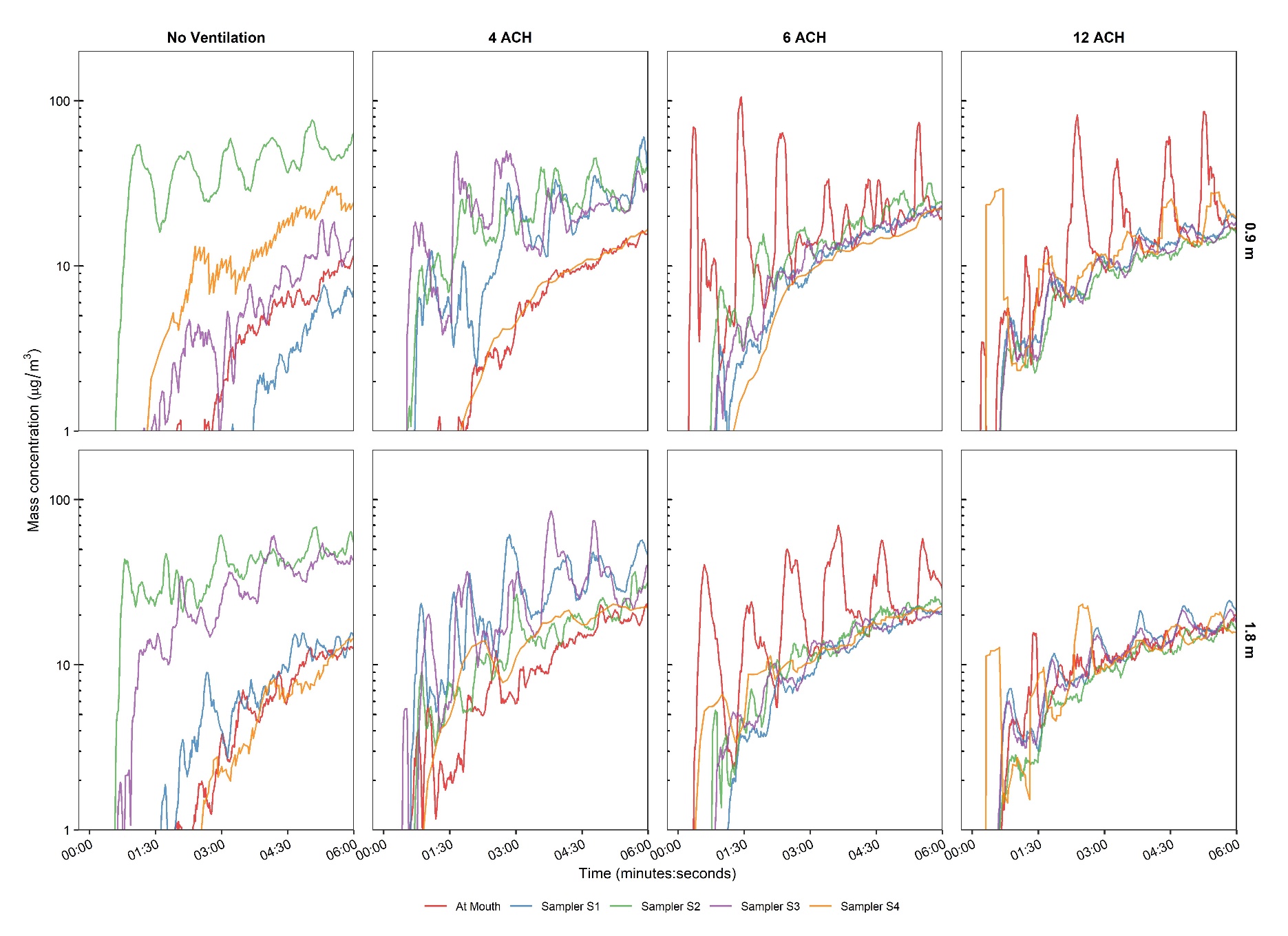


Supplemental Figure S6. OPC-specific Time Concentration Curves for Unmasked Breathing. Mean chamber mass concentration time curves for the first 6 minutes of exposure of simulated very fine respiratory droplets and aerosol particles produced by breathing for the examined physical distance and ventilation rates. The OPC time concentration curves designated beside the head of the recipient for the unmasked scenarios was similar to the mouth of the breather and, thus, omitted for clarity. ACH = Air changes per hour.


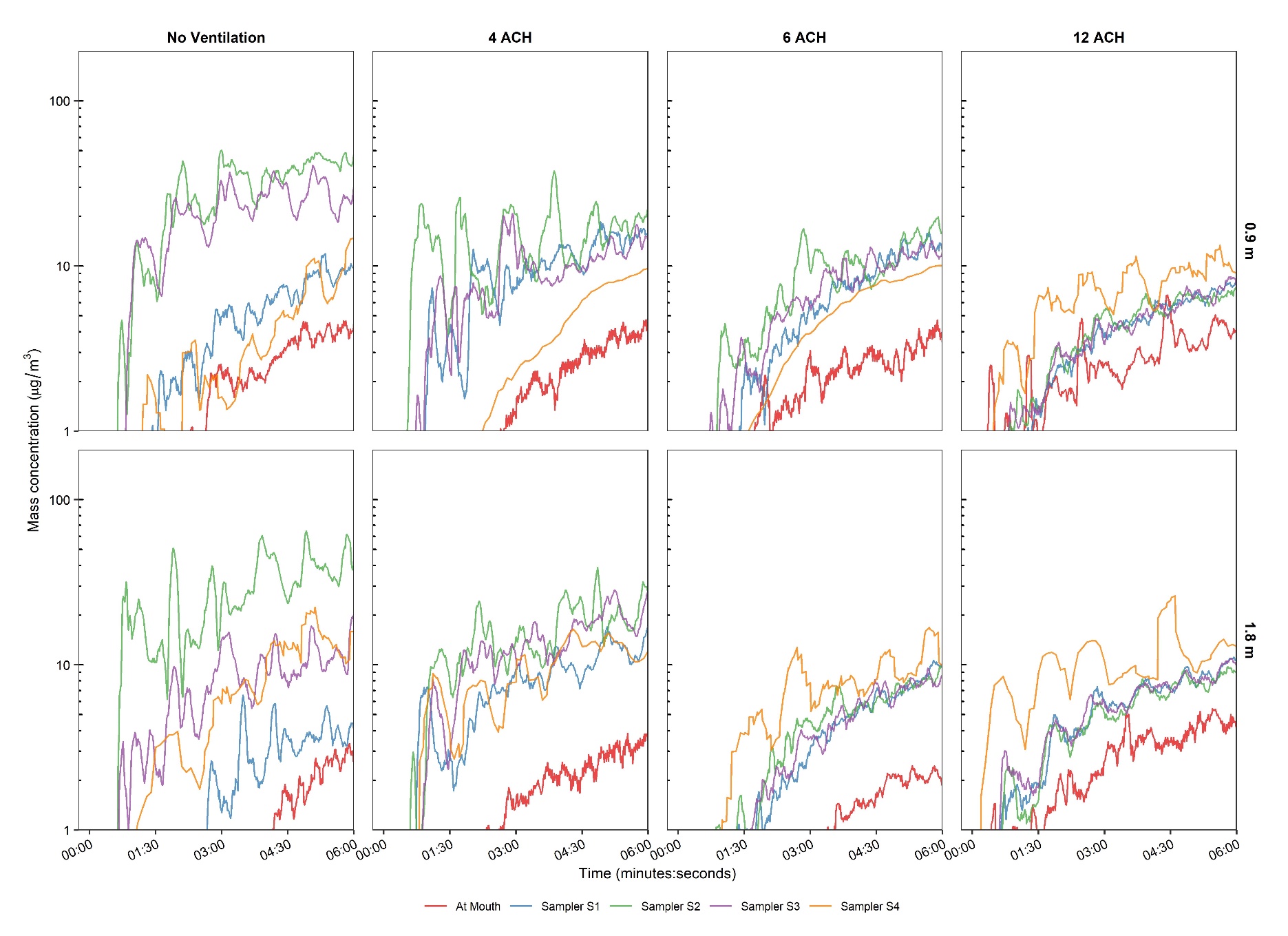


Supplemental Figure S7. OPC-specific Time Concentration Curves for Universal Masked Breathing. Mean chamber mass concentration time curves for the first 6 minutes of exposure of simulated very fine respiratory droplets and aerosol particles produced by breathing for the examined physical distance and ventilation rates. The OPC time concentration curves designated beside the head of the recipient for the unmasked scenarios was similar to the mouth of the breather and, thus, omitted for clarity. ACH = Air changes per hour.


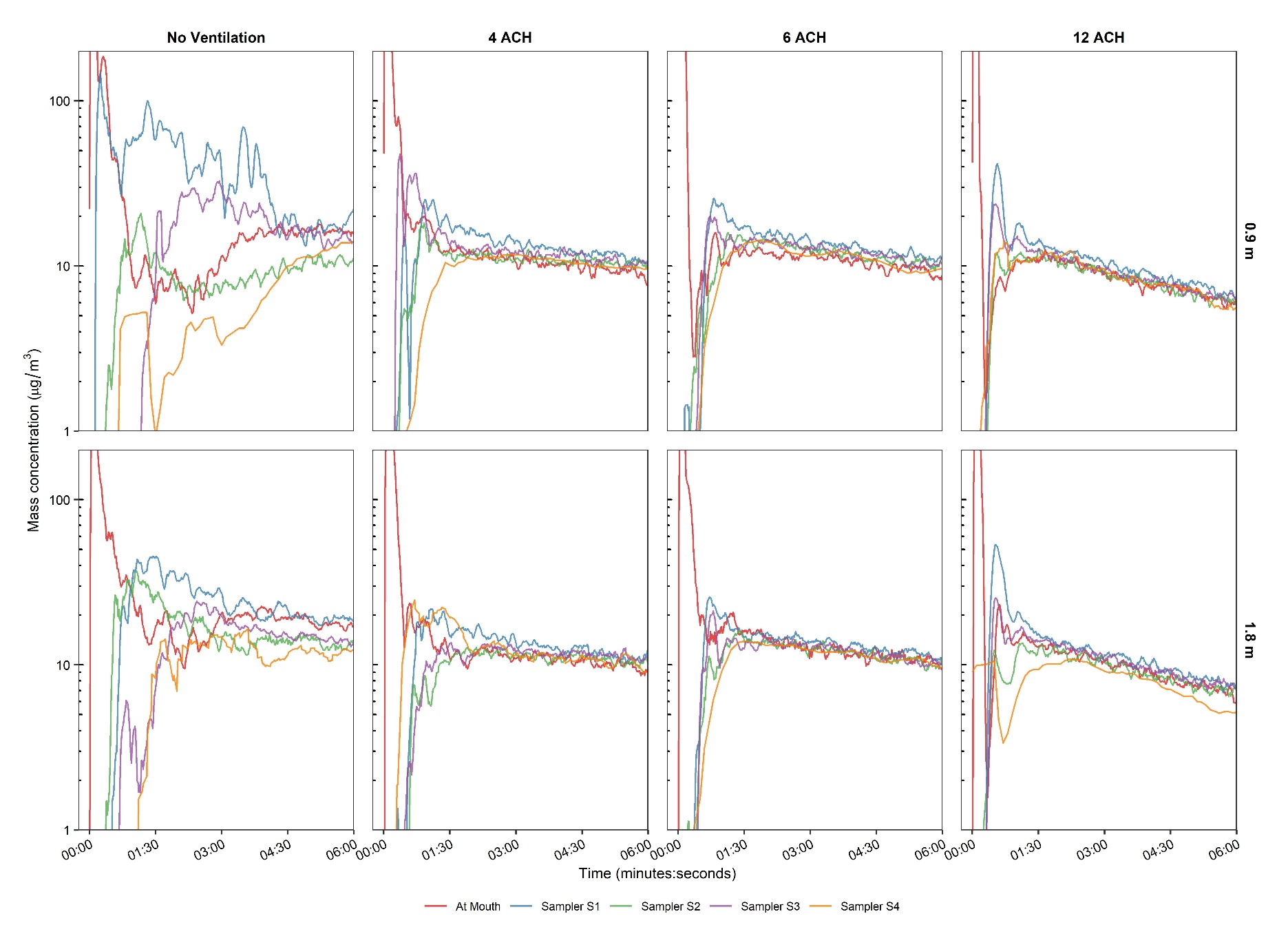


Supplemental Figure S8. OPC-specific Time Concentration Curves for Unmasked Single Cough. Mean chamber mass concentration time curves for the first 6 minutes of exposure of simulated very fine respiratory droplets and aerosol particles produced by breathing for the examined physical distance and ventilation rates. The OPC time concentration curves designated beside the head of the recipient for the unmasked scenarios was similar to the mouth of the breather and, thus, omitted for clarity. ACH = Air changes per hour.


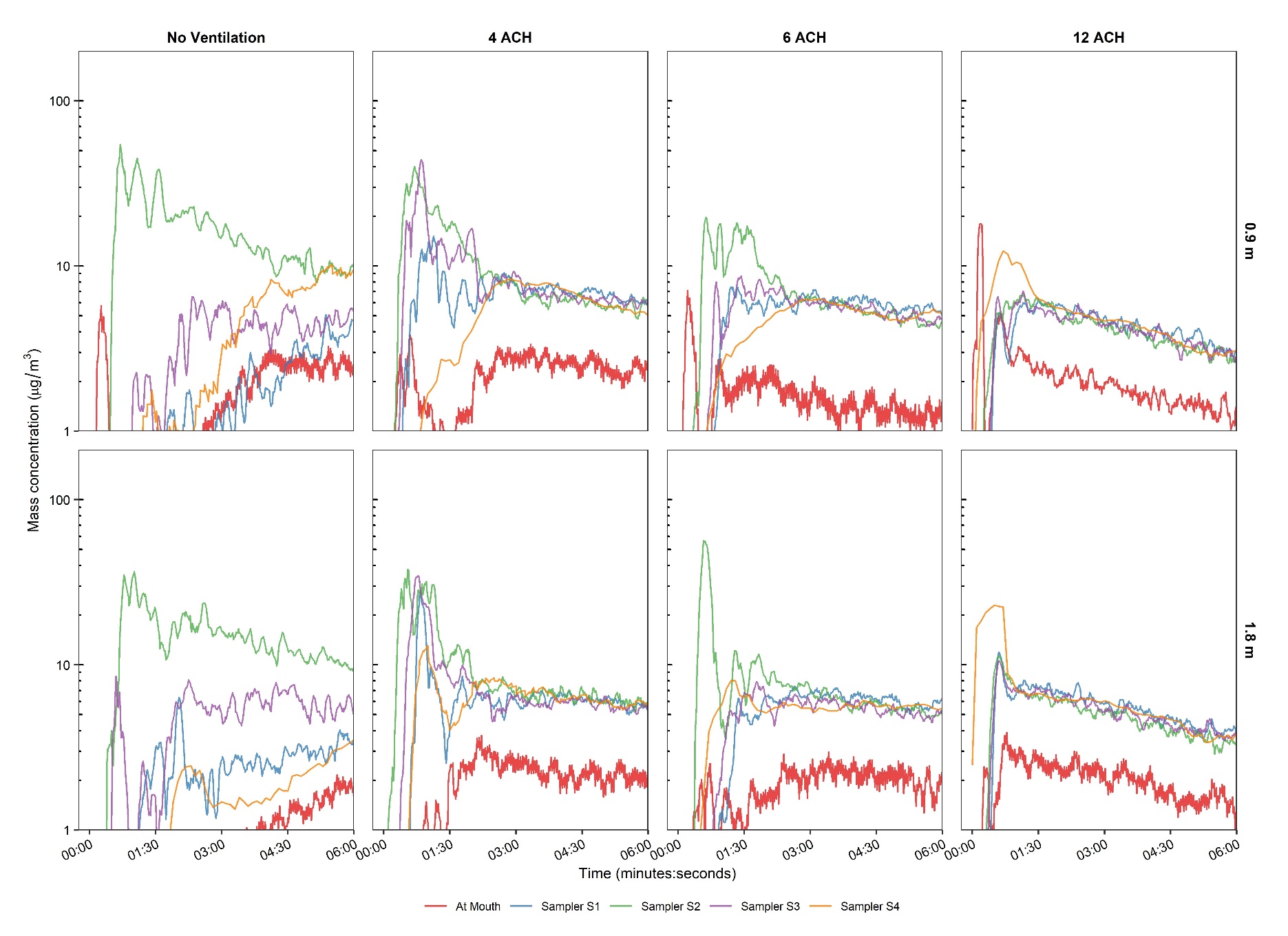


Supplemental Figure S9. OPC-specific Time Concentration Curves for Universal Masked Single Cough. Mean chamber mass concentration time curves for the first 6 minutes of exposure of simulated very fine respiratory droplets and aerosol particles produced by breathing for the examined physical distance and ventilation rates. The OPC time concentration curves designated beside the head of the recipient for the unmasked scenarios was similar to the mouth of the breather and, thus, omitted for clarity. ACH = Air changes per hour.

| **Supplemental Table 1. Regression Coefficients for Short-term Aerosol Generation Test** | | | | | | | | |
| --- | --- | --- | --- | --- | --- | --- | --- | --- |
| **Modality** | **Parameter** | **Regression Coefficients** | | **Percent Reduction** | | **t-value** | **Pr > \|t\|** | **Model Adjusted R^2^** |
|  |  | **β** | **CI95%** | **Estimate (%)** | **CI95%**  **(%)** |  |  |  |
| Short-term Aerosol Generation | Constant | 3.923 | 3.792 to 4.054 | - | - | 60.394 | < 0.001 | 0.933 |
|  | Distance: 1.8 m | -0.050 | -0.172 to 0.072 | 4.9 | -7.4 to 15.8 | -0.829 | 0.412 |  |
|  | ACH | -0.053 | -0.067 to -0.039 | 5.2 | 3.8 to 6.5 | -7.577 | < 0.001 |  |
|  | Masking: Yes | -1.477 | -1.599 to -1.355 | 77.2 | 74.2 to 79.8 | -24.423 | < 0.001 |  |
| ACH = Air changes per hour | | | | | | | | |
